# Cylindrical Multimode Waveguides as Focusing Interferometric Systems

**DOI:** 10.1021/acsphotonics.2c02030

**Published:** 2023-05-17

**Authors:** Wladislaw Michailow, Nikita W. Almond, Harvey E. Beere, David A. Ritchie

**Affiliations:** † Cavendish Laboratory, University of Cambridge, JJ Thomson Avenue, CB3 0HE Cambridge, United Kingdom; ‡ Department of Physics, Swansea University, Singleton Park, Sketty, SA2 8PP Swansea, U.K.

**Keywords:** terahertz optics, lenses, imaging, Talbot effect, multimode interference, self-imaging

## Abstract

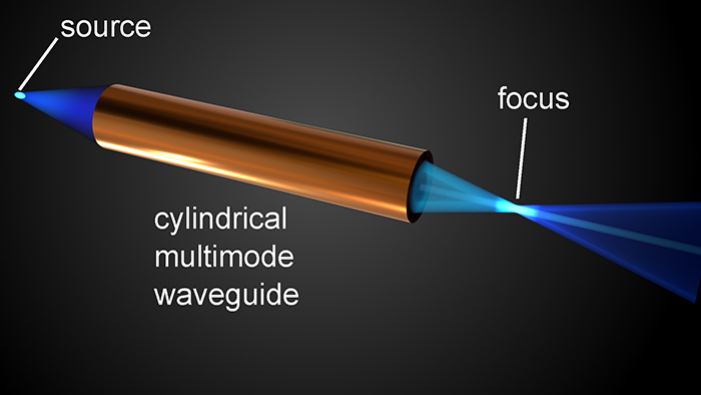

Delivery and focusing of radiation requires a variety of optical elements such as waveguides and mirrors or lenses. Heretofore, they were used separately, the former for radiation delivery, the latter for focusing. Here, we show that cylindrical multimode waveguides can both deliver and simultaneously focus radiation, without any external lenses or parabolic mirrors. We develop an analytical, ray-optical model to describe radiation propagation within and after the end of cylindrical multimode waveguides and demonstrate the focusing effect theoretically and experimentally at terahertz frequencies. In the focused spot, located at a distance of several millimeters to a few centimeters away from the waveguide end, typical for focal lengths in optical setups, we achieve a more than 8.4× higher intensity than the cross-sectional average intensity and compress the half-maximum spot area of the incident beam by a factor of >15. Our results represent the first practical realization of a focusing system consisting of only a single cylindrical multimode waveguide, that delivers radiation from one focused spot into another focused spot in free space, with focal distances that are much larger than both the radiation wavelength and the waveguide radius. The results enable design and optimization of cylindrical waveguide-containing systems and demonstrate a precise optical characterization method for cylindrical structures and objects.

## Introduction

Delivery of electromagnetic radiation from one point to another is generally accomplished either using free space optics or using waveguides. In the microwave range, antennas are used to send and to receive electromagnetic signals wirelessly, and metallic single-mode waveguides are a common tool used for waveguide-coupled systems. In infrared and visible optics, lenses and parabolic mirrors focus and collimate free-space radiation, while single-mode and multimode optical fibers are successfully used to guide radiation over long distances and curved paths.

In the terahertz (THz) region, the majority of measurements are carried out using free-space optics, utilizing components such as lenses or parabolic mirrors. This approach has several drawbacks when working in the THz regime: experiments often require a purged gas environment or radiation delivery to difficult-to-access spaces, such as cryostats. To achieve the tightest focal spot incident on a sample, a high numerical aperture is needed,^1^ which requires a small focal distance or large optical elements, which often cannot be accommodated. Additionally, this configuration complicates the quantification of the incident intensity and makes the setup prone to small variations in the focal position. A beam of low divergence requires a low numerical aperture, which results in a larger spot size and makes focusing prone to angular misalignment.^2^ The optical components are expensive, and their bulkiness complicates usage in scenarios where a purged environment is required.

These issues could be eliminated by the use of waveguides. However, in the THz range, a number of obstacles hinder their exploitation to date. Single-mode waveguides, which have the advantage of delivering one waveguide mode with a clearly defined polarization and mode profile, become increasingly impractical at higher frequencies,^3^ due to prohibitively high losses,^4,5^ difficulty in coupling and fabrication, and high cost. In dielectric terahertz fibers, losses per length are substantially higher than in their optical counterparts.^6^ In a straight, hollow cylindrical waveguide, the attenuation of the (*l*, *m*)-mode can be described as
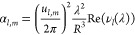
1where λ is the wavelength and *R* is the radius of the waveguide.^7,8^*u*_*l*,*m*_ is the *m*-th zero of the Bessel function *J*_*l*–1_(*x*), and Re(ν_*l*_(λ)) is a parameter dependent on the mode, waveguide geometry, and complex refractive index of wall material.^7^ The 1/*R*^3^-dependence of losses for a given mode makes multimode waveguides with a large radius, *R* ≫ λ, attractive.

Mixing of modes in the multimode waveguide may result in a large size of the output beam with a speckle pattern at the output. In the area of imaging with multimode fibers, researchers have learned to make use of the resulting speckle patterns at one end of a fiber by reconstructing images sent through the fiber from its other end.^9−12^ Such experiments usually involve coupling radiation into and out of a multimode fiber using a microscope objective or lens at both ends and detecting the speckle pattern of the transmitted light through the fiber by an imaging CCD array, reconstruction of the transfer matrix of the fiber by sending many known images through the fiber, which may involve machine learning approaches with neural networks, and finally calculation of the original image based on the characterized properties of the multimode fiber.^9−12^ However, if the goal is to focus radiation after a multimode waveguide, a speckle pattern represents a poor profile, which will be impractical for further focusing by lenses or parabolic mirrors.

To achieve a defined beam pattern at the output of multimode waveguides, precise control over mode interference is necessary. Multimode interference can be used to realize various devices, e.g., to split^13^ and combine beams,^14^ and realize power dividers.^15^ Photonic crystal waveguides enable realization of integrated versions of such systems.^16−18^ Mode interference has also formed the basis for beam-steering applications in antenna arrays.^19^ Mode converters and launchers are used to couple between waveguide and free space modes or different types of waveguide modes.^20^ For example, in gyrotrons, quasi-optical mode converters are used to convert higher-order waveguide modes of gyrotron oscillators into a Gaussian field distribution.^21^ A commonly used type of mode converters is the Vlasov launcher,^22^ which consists of a stepped-cut cylindrical Vlasov launcher, an elliptical and a parabolic reflector, and can be designed using ray-optical calculations.^23^

The first observation of multibeam interference for focusing was done by Talbot in 1836:^24^ if a transmission grating with holes is placed after an object, the image of the object is reproduced after the grating at multiple positions, a phenomenon called the Talbot effect. Thus, a grating with a two-dimensional array of holes can be seen as a focusing optical element with multiple “focal positions”, where the image is reproduced.^25,26^ In multimode waveguides, the Talbot effect^27^ is referred to as self-imaging:^28^ due to the reflective boundary conditions at the waveguide walls, each point within a multimode waveguide has a corresponding set of points where the wavefield is reproduced or “revived”. Initial demonstrations were done on planar, or rectangular, waveguides.^29^ It has been shown that the resolution of the self-imaging effect in planar waveguides is about , which is much smaller than the waveguide width *W*.^30^ This enables multimode waveguides to be used as focusing systems.^31,32^ In planar waveguides, waveguide grating lenses have been used for focusing of visible light.^33^ Self-imaging in cylindrical waveguides was theoretically analyzed in ref (34). In the area of THz research, the Talbot effect has been realized on a grating^26^ and for passive pulse amplification in the temporal domain.^35^

In our work, we exploit multimode interference and the self-imaging phenomenon in cylindrical multimode waveguides for focusing. We will show that a cylindrical waveguide itself can serve as a focusing element providing a tight spot at its output, without any external lenses, parabolic mirrors, or microscope objectives. The need to eliminate any external focusing elements is particularly strong in use cases where due to space contraints the bulkiness and free-space path length of focusing optics becomes prohibitive and can make measurements impossible. This issue arises especially in cryogenic environments, i.e. in cryostats and dilution refrigerators, that have very small sample spaces, and is further complicated by the requirement for a vacuum environment, low temperatures, and proper thermal contact for cooling of all components in such systems. At the same time, these constraints present obstacles to in situ alignment of the optical path using e.g. tip-tilt stages–a problem that does not arise in a benchtop optical system in ambient air. This is one of the reasons why in the THz range the use of multimode cylindrical waveguides is on the rise as a radiation delivery system to cryogenic environments.^36,37^ Apart from cryogenic environments, using a multimode waveguide as the one and only element for both focusing and radiation delivery opens the door to a variety of endoscopic applications for material probing and coating inspection^38^ inside of objects, in deep cavities accessible only through a small hole, e.g., in car or aircraft engines or for medical endoscopy and chirurgic operations in humans or animals.

In this work we show how cylindrical multimode waveguides can be used to focus radiation to a tight spot. We present a fully analytical description of radiation propagation in a cylindrical multimode waveguide within a ray-optical treatment. Our model allows calculation of the wave propagation and positions of maxima not only within the multimode waveguide along its length, but also of the beam profile in the free space after the waveguide. We show how to design a waveguide to exhibit focusing after its end, with an intensity significantly exceeding the intensity at the input of the waveguide. We experimentally demonstrate the focusing at terahertz frequencies using radiation from a 1.9 THz quantum cascade laser guided through a cylindrical hollow-metal waveguide with ∼4.6 mm diameter, while simultaneously benefiting from the low THz losses of the waveguide due to its multimode nature. We show how the waveguide achieves a focused spot several millimeters to a few centimeters away from the waveguide end, which covers typical focal distances in optical setups, thereby replacing two lenses or parabolic mirrors. The findings represent the first practical realization of a focusing system consisting of only a single cylindrical multimode waveguide, that delivers radiation from one focused spot Q into another focused spot P in free space with focal distances that are both much larger than the radiation wavelength and much larger than the waveguide radius.

## Theory

**Figure 1:**
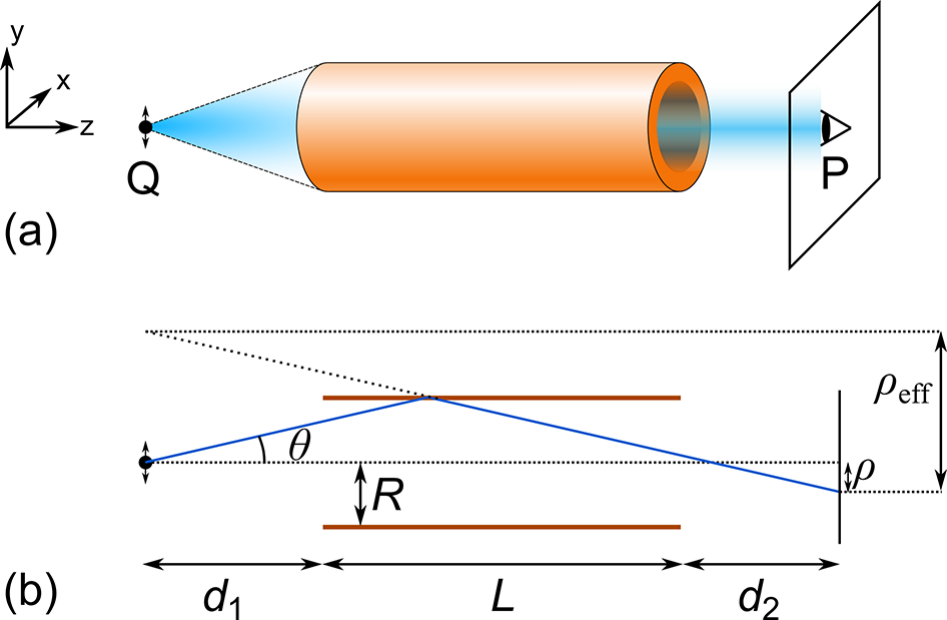
Ray-optical model. (a) Source Q emits radiation predominantly in the *z*-direction along the axis of the hollow metal cylindrical waveguide and hits a screen at point P. (b) Distance and variable definitions for the ray paths shown in the radial cross section of the waveguide. ρ is the actual radius on the screen, and ρ_eff_ is the total distance traveled perpendicular to the *z*-axis.

To understand the nature of radiation propagation within a multimode cylindrical waveguide, we calculate the expected beam profile within a ray optical treatment. We consider a coherent source located at the point Q, which represents a THz quantum cascade laser (QCL),^39^ that emits radiation into the waveguide, where it propagates until it hits the observer at point P. At this point a screen is located to measure the electric field, see Figure 1. The distance from the source Q to the waveguide input is *d*_1_; the separation between the waveguide output and the screen is *d*_2_. The waveguide is assumed to be lossless and straight, has a length *L* and a radius *R*. We consider the case of a large multimode waveguide with *R* ≫ λ. This condition justifies the use of the ray-optical treatment. The waveguide axis is in the *z*-direction, and the source Q lies on the same axis, i.e., we consider meridional rays, but not skew rays.

Rays emitted by the source at Q may go straight through the waveguide, without reflections, or be reflected at the waveguide walls once, twice, or more. To calculate the resulting electric field at the screen, the electric fields of all rays incident at a point (*x*, *y*) at the screen are summed up.

**Figure 2:**
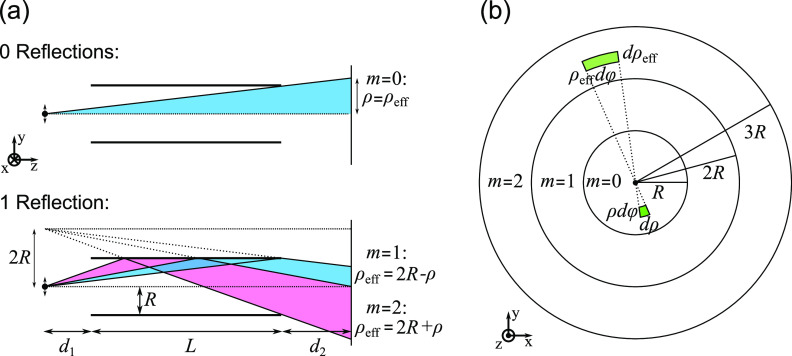
Ansatz to calculate the individual rays. (a) Ray paths and beam orders *m* for rays experiencing zero and one reflections. (b) Image seen by an observer at P looking into the waveguide along its axis. The rays seem to come from imaginary annuli around the central circle of radius *R*. Energy conservation leads to the intensity amplification factor ρ_eff_/ρ for reflected rays.

The radial coordinate on the screen is . The total distance traveled by a ray in lateral directions (*x* and *y*) is ρ_eff_. For the ray that goes straight through, which we will label with number *m* = 0, we have ρ_eff_ = ρ, see Figure 2a. For any rays that have been reflected at the walls of the cylindrical waveguide, ρ_eff_ is larger than ρ, and their relationship depends on the number of reflections and can be derived geometrically as shown in Figure 2 (a). For *m* = 1, ρ_eff_ = 2*R* – ρ, the ray experiences one reflection, but still ends up above the *z*-axis on the screen. The case *m* = 2 corresponds to one reflection that ends up below the *z*-axis; since ρ > 0 always, this corresponds to a flip of ϕ → ϕ + π in the wavevector *k⃗*, with ρ_eff_ = 2*R* + ρ. In general,
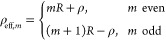
2For an observer at P, rays with indices *m* > 0 seem to originate from an imaginary annulus around the ring of radius *R* at the position *z* = *d*_1_ of the waveguide input, see Figure 2b. Each ray of index *m* effectively originates from an annulus bound by circles of radii *mR* and (*m* + 1)*R*, and experiences *N*_refl_ = ⌊(*m* + 1)/2⌋ reflections, where ⌊·⌋ indicates the floor function.

The total traveled distance *r*_eff,*m*_ of ray *m* is
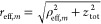
3where the total distance traveled in the *z*-direction is *z*_tot_ = *d*_1_ + *L* + *d*_2_. The rays will travel at an angle θ_*m*_ with respect to the *z*-axis, which gives
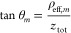
4The source Q is modeled as a Hertzian dipole  parallel to the *y*-axis, which emits an electric field *u* in the far field. Its angular intensity distribution is described by a function *f*(θ, ϕ):
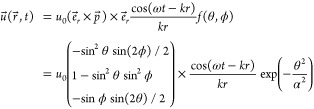
5To describe the enhanced emission probability of a QCL in the solid angle around the *z*-axis, with angles 0 ≤ θ ≲ α, we used here *f*(θ, ϕ) = exp(−θ^2^/α^2^).

Summing up contributions from all rays we finally get the electric field at the point (*x*, *y*), as we will explain in the following:
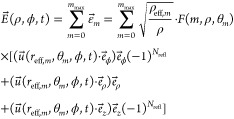
6The magnetic field is
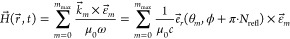
7The intensity *I*(*x*, *y*) impinging on the detection plate located at a distance *d*_2_ from the waveguide end is then calculated as the time-averaged *z*-component of the Poynting-vector .

In eq 6, the individual electric fields are multiplied by a conditional function *F*(*m*, ρ_*m*_, θ_*m*_), that is either 1 or 0, and a “density of states”-type factor . When a ray is reflected at the cylindrical hollow metal waveguide, conservation of the parallel components of the electric field requires the reflected wave to have a phase flip of π or a sign flip. Thus, the electric field is decomposed in the cylindrical coordinate system, and the parallel ϕ- and *z*-components gain a sign flip of . Similarly in the H⃗-field, reflections flip the vector *k⃗* of each ray antisymmetrically with respect to the *z*-axis, which adds π to ϕ whenever the number of reflections is odd.

The conditional function *F*(*m*, ρ, θ_*m*_) tells when a ray has to be considered in the sum:
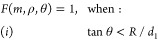
8(only rays that entered the waveguide are considered)
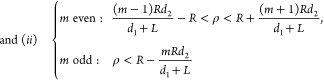
9(condition when an *m*-th order beam reaches the screen)
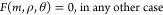


Using the first condition (eq 8) as well as eqs 2 and 4, the number *m*_max_ of terms in the sum to be considered can be derived to be *m*_max_ = ⌈*L*/*d*_1_⌉, where ⌈·⌉ denotes the ceiling function.

The factor  in eq 6 can be understood as follows, see Figure 2b: All rays that would have illuminated the annular segment ρ_eff_dϕdρ_eff_ on the screen if they were not reflected (i.e., if there was no waveguide), actually end up on the screen in the annular segment ρdϕdρ. Since the power contained within the segments must be the same, *I*_0_|ρ_eff_dϕdρ_eff_| = *I*_1_|ρdϕdρ|, it follows that *I*_1_/*I*_0_ = ρ_eff_/ρ, and its square root accounts for this in the sum of the electric fields, eq 6.

This leads to a ∼ 1/ρ-divergence of the intensity at the center of the waveguide. It demonstrates the focusing ability the waveguide. A conventional spherical lens focuses a 2D collimated beam onto a 0D spot, with a ∼ 1/ρ^2^-divergence in the focal spot. A cylindrical lens focuses a 1D collimated line onto a 0D spot in its focal plane, with a ∼1/ρ-divergence of the intensity. In the case of the multimode cylindrical waveguide, a 1D circle circumference is focused onto the spot at ρ = 0. A remarkable property is that even in the case of incoherent light, cylindrical waveguides exhibit focusing of an incident beam onto the axis due to the  factor, an effect that is not expected for rectangular or planar waveguides.

Our theoretical analysis considers a lossless waveguide. In the case of Ohmic losses in the metal of the waveguide, an additional factor should be included. For a ray undergoing *N*_refl_ reflections, there will be a reduction in amplitude at each reflection, and in the case of complex reflection, a phase shift. Such a factor, ζ, will enter formula eq 6 as a *k⃗*-dependent and *N*_refl_-dependent value, as .

**Figure 3:**
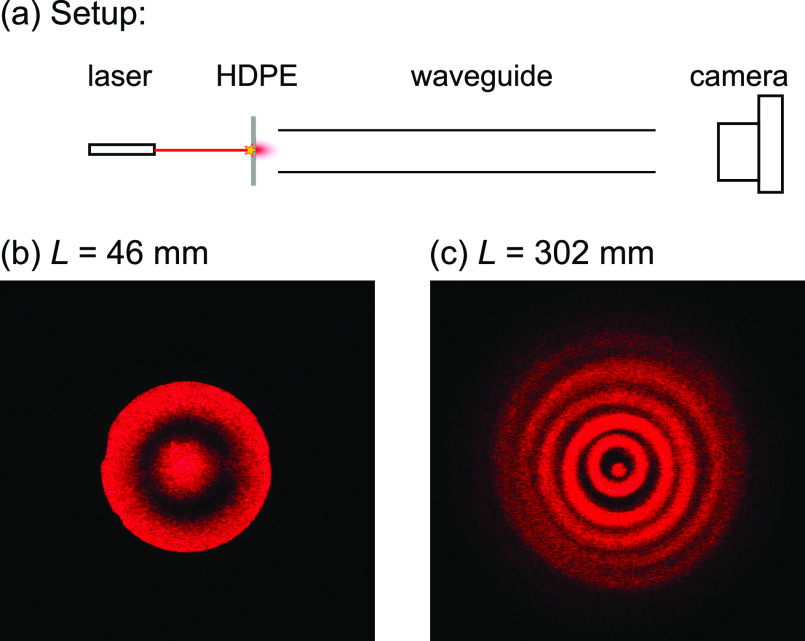
Circular rings in the visible range. (a) Setup to capture the output of a multimode waveguide with a camera using collimated red light from a HeNe laser in the visible range (632.8 nm wavelength). (b, c) Photos at the waveguide output for different waveguide lengths *L* of 46 (b) and 302 mm (c). Image (c) reveals the imperfections of the real waveguide: the rings are not perfectly concentric, and some are slightly ellipsoidal.

Interestingly, the circles of increasing radius shown in Figure 2b are not a purely theoretical construct, but can be displayed to the eye in the visible range. This is shown in Figure 3a. A red laser spot from a helium–neon laser, emitting at 632.8 nm, is incident on a high-density polyethylene (HDPE) sheet, which acts as a diffusor, at the start of a cylindrical hollow-metal waveguide of ∼4.6 mm diameter. The spot hits the HDPE sheet at the center of the waveguide, i.e., can be seen as a radial source on the waveguide axis. For the wavelength of the red laser radiation, this waveguide is a multimode waveguide. The image seen at the end of the waveguide is captured by a camera, yielding Figure 3b,c for two different lengths of the waveguide, 46 and 302 mm. Indeed, circular rings of increasing radius can be observed. More rings are seen for the longer length, as in this case rays undergo more reflections. The number of reflections, *N*_refl_ = ⌊(*m* + 1)/2⌋, increases with *L* as *m*_max_ = ⌈*L*/*d*_1_⌉, and those rays within the solid angle of the camera lens aperture are captured. Not all rings are concentric, and some are slightly ellipsoidal, which highlights the imperfections of the real waveguide.

Using the ray-optical model it is not possible to predict the achievable resolution. In reality, the electric field will not have singularities, since diffraction restricts the resolution to a value which can only be derived from wave optics, on a length scale of λ. Experimentally, how tight the focus will be is limited by the dimensions of the source and the aperture of the detector. Therefore, we will convolve the resulting intensity distribution with a power-conserving Gaussian function
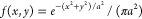
10with an averaging constant *a*,
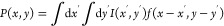
11in order to obtain physically sensible mode profiles *P*(*x*, *y*).

The ray-optical treatment is valid in the case of *R* ≫ λ, a condition that is fulfilled here as multimode cylindrical waveguides are considered. Also, for distances *d*_2_ ≫ 2*R*, that are much larger than the diameter after the waveguide, the accuracy of the results starts to degrade, as scattering effects on the edges of the waveguide end become significant.

**Figure 4:**
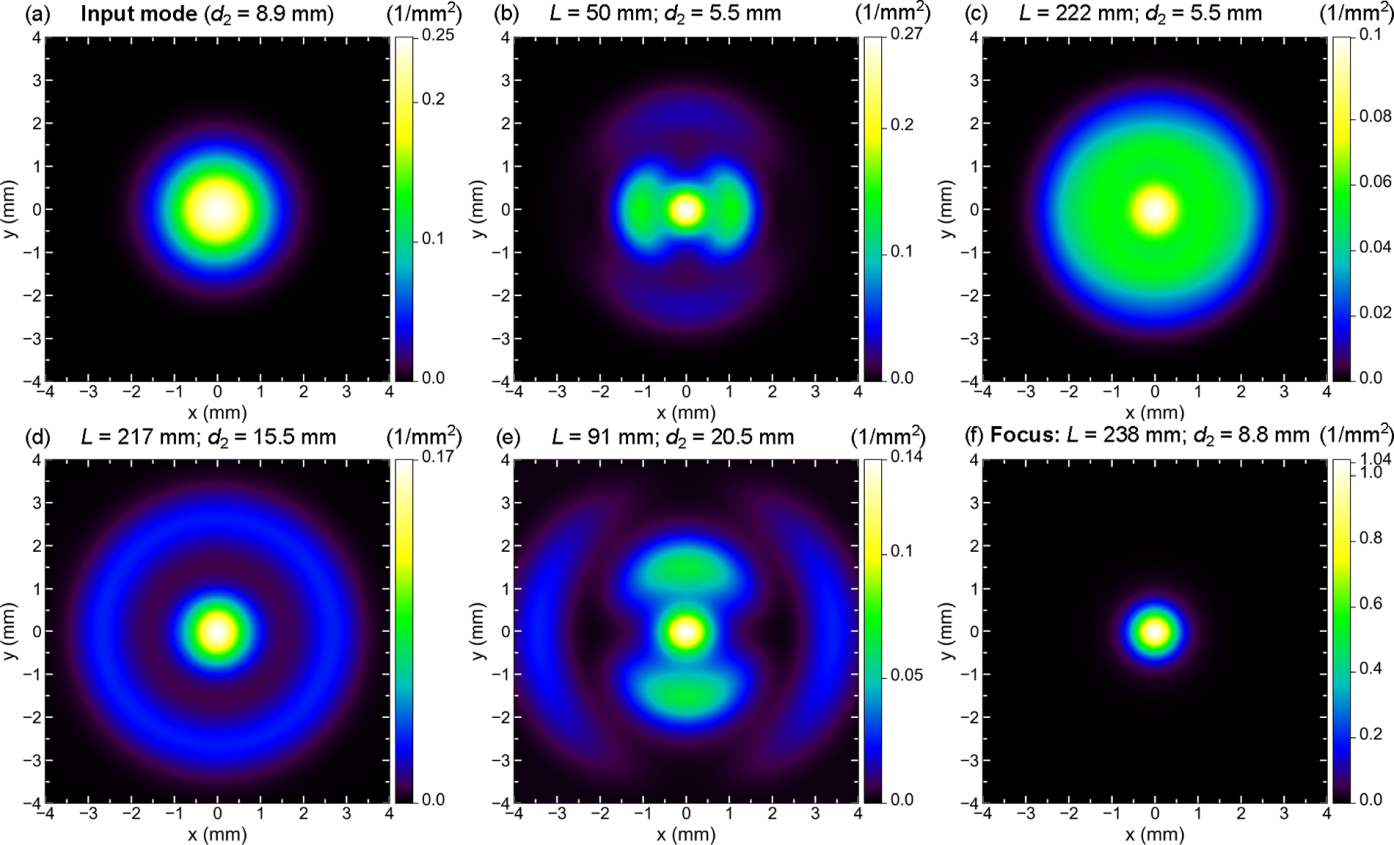
Theoretically predicted input and output mode profiles, at a wavelength of λ = 159.38 μm. The intensity in 1/mm^2^, averaged with a Gaussian with FWHM of 0.78 mm and normalized to a total integrated power of 1, is shown as a color map. The value at the top of the color bar shows the maximum intensity for a graph. (a) Input mode profile, i.e., the intensity distribution at the waveguide input plane. (b–f) Output mode profiles at various waveguide lengths *L* and distances *d*_2_ between waveguide end and detector plane, with a source-waveguide distance of *d*_1_ = 8.9 mm. (f) shows the output mode profile 8.8 mm after the end of a 238 mm waveguide, where optimal focusing is achieved, this will be called the “focus position”.

The ray-optical model allows us to calculate the expected mode profiles along the length of the waveguide and after its end. Figure 4 shows exemplary mode profiles for values of *R* = 2.268 mm radius, and *d*_1_ = 8.9 mm distance between the source and the waveguide entry point at a wavelength of λ = 159.38 μm and an angle α = 9.5° (parameters used in the experimental section below). The mode profiles are averaged using a Gaussian with a full width at half-maximum (FWHM) of 0.78 mm, which corresponds to  mm in eq 10 and best mimics the experimental resolution of a 1 mm aperture used in the experimental section. The intensity is normalized to the total integrated power. It should be noted that the color scale is normalized to the maximum intensity in each individual graph, with the maximum value shown at the top of the color bar.

The input mode profile at the entrance of the waveguide can be seen in Figure 4a. The color map indicates the intensity values. As expected, a Gaussian-type input mode profile that is cut off at the radius of the waveguide can be seen.

Now this electric field distribution propagates through the waveguide, and for the exemplary values given above, we plot the theoretically calculated output mode profiles in Figure 4b–f for different lengths *L* of the waveguide and different distances *d*_2_ between the waveguide end and the detector. Common to the graphs is a central spot originating from the focusing ability of the cylindrical waveguide. Depending on the length of the waveguide, the output mode profile can show circular rings as in (c) and (d), or sidelobes, as in (b) and (e). With increasing distance from the waveguide end, the rings/cones or sidelobes move further out from the center. Under some conditions, which we will discuss in the following, the output mode can be a tightly focused spot, as seen in Figure 4f, with a peak intensity significantly exceeding the maximum intensity at the waveguide input plane.

**Figure 5:**
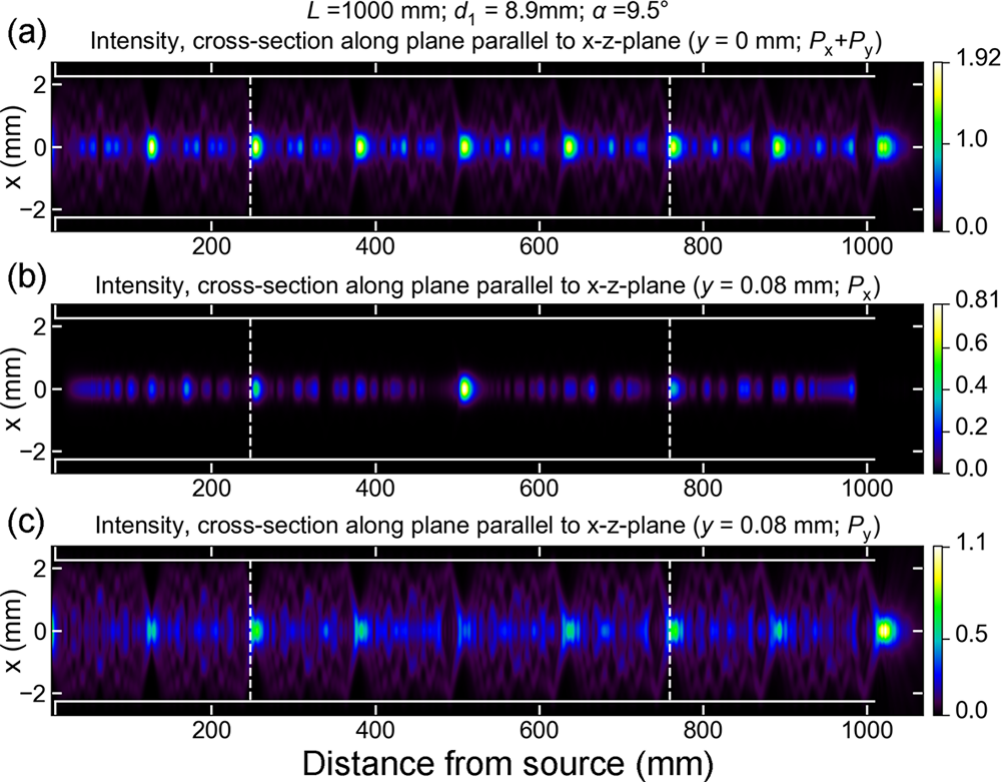
Wave propagation within the waveguide. Cross-sectional cuts showing the intensity, radially averaged over *a* = 2λ, as described in Appendix A. Power generated by the *x*- and *y*-components of the incident electric field in the *x*–*z*-plane (a); Power generated by the *x*-component (b) and *y*-component (c) of the incident electric field in a plane parallel to the *x*–*z*-plane shifted by λ/2. A periodicity is observed, which is indicated by the white dashed lines.

To understand the origin of the mode profiles, we show their evolution within the waveguide over a large waveguide length *L* = 1 m. It is shown as a cross section along the *x*-axis, see Figure 5. To illustrate the mode profiles in the *x*–*z*- and *y*–*z*-planes, we use an approximative one-dimensional averaging approach as described in Appendix A. In Figure 5a, the total power *P*_*x*_ + *P*_*y*_ originating from *x*- and *y*-polarized light is shown. Here, *P*_*x*_ + *P*_*y*_ is equal to *P*_*y*_ only, since there is no electric field in the *x*-direction along the *x*- and *y*-axes for symmetry reasons. To illustrate the evolution of the *x*- and *y*-polarized electric field, in Figure 5b,c, the *P*_*x*_ and *P*_*y*_ powers are shown in a plane parallel to the *x*–*z*-plane, shifted by *y* = 0.08 mm ≈ λ/2. The mode profile repeats itself periodically. While at symmetry planes shorter periods are observed, the longest period can be seen in the *P*_*x*_-diagram, approximately 516 mm, and is indicated by the white dashed lines.

Since the input electric field is predominantly *y*-polarized due to the *y*-orientation of the Hertzian dipole in the model, the *P*_*x*_ component in Figure 5a is initially zero, until the interference in the waveguide sets in. The multimode interference in the waveguide mixes polarization states and leads to the generation of an *x*-polarized electric field at certain positions along the length of the waveguide of a much larger magnitude than what is present in the input electric field. This phenomenon has its origin in the sign flip of the -component that the electric field experiences when it is reflected at the walls of the cylinder, see eq 6. If the sign flip of the -component is not considered, the model would predict nearly symmetric output mode profiles.

Within a distance *d*_2_ ≲ *d*_1_ after the waveguide, the axial beam pattern follows the mode profile within the waveguide. To obtain a focused central spot at a distance *d*_2_ ≲ *d*_1_ after the end of the waveguide, the waveguide has to be cut to a length *L* = *z*_max_ – *d*_2_, where *z*_max_ is the *z*-position of a maximum along the waveguide axis.

**Figure 6:**
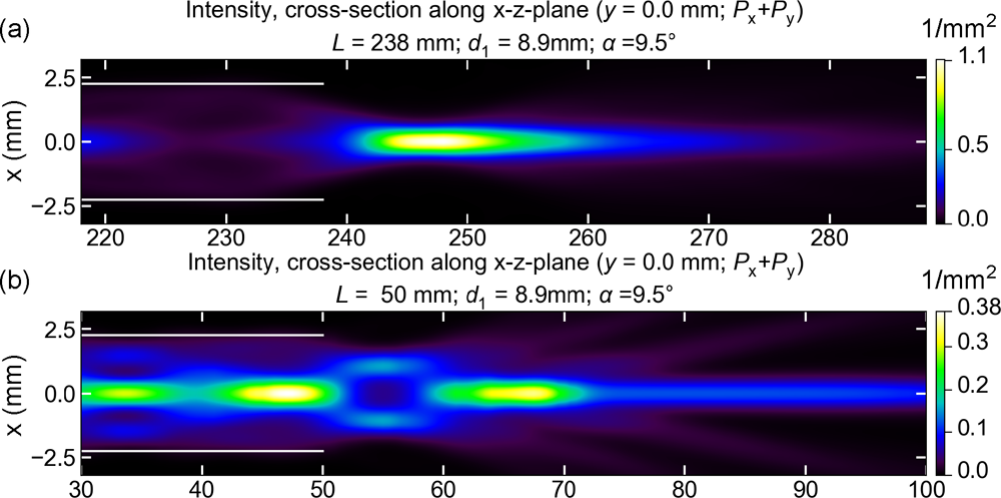
Focusing effect of the multimode waveguide. (a) Mode pattern propagation after the waveguide end if the waveguide is cut at *L* = 238 mm, ca. 8.8 mm before the second maximum, at the first dashed line in Figure 5. Optimal focusing is achieved after the waveguide, corresponding to an effective “focal distance” of 8.8 mm. (b) Mode propagation for a waveguide length *L* = 50 mm. The focusing is less optimal, but the maximum intensity is still achieved along the waveguide axis.

For example, we consider the second clearly visible maximum position in Figure 5a for focusing at the end of the waveguide. In Figure 5 the waveguide is “cut” at *L* = 238 mm, indicated by the first white dashed line, and the behavior at the output of the waveguide is shown in cross section in Figure 6a. A clear focal spot is obtained at 8.8 mm after the end of the waveguide, demonstrating the focusing ability of the waveguide. For comparison, the output mode profile is also shown for a length *L* = 50 mm in Figure 6. Here, the output intensity distribution is less well focused. While there is still a peak on the waveguide axis, its intensity is about three times smaller, and much more power is contained in side-lobes or cones that separate themselves and move outward, away from the axis. Notably, the output beam is not Gaussian; it has a complicated structure originating from multiple beam interference. The divergence is low: over a distance of 2 cm, most of the power is still contained within a radius of 3 mm.

Similarly, if a length *L* of a waveguide is given, the above calculation can be used to answer the question, how far away from the waveguide input should the source be positioned, to obtain maximal central intensity at a given distance *d*_2_ from the waveguide end.

## Experimental Realization

As we have shown, multimode waveguides can be used not only to guide, but also to focus terahertz radiation, and thanks to their large diameter, the terahertz losses are low. We now cut a waveguide of length 238 mm to check our theoretical predictions, and measure its performance as a THz focusing element.

To this end we built an experimental setup according to the diagram shown in Figure 1. The THz source we use is a single-plasmon quantum cascade laser, emitting at a wavelength of 159.38 μm (≈ 1.9 THz), that is cooled to 18 K in a continuous-flow liquid helium cryostat. The QCL design is the same as in ref (40), but with 4.4 nm instead of 5.0 nm injection barrier thickness. The copper waveguide used is made out of annealed copper and has an inner diameter of (4.6 ± 0.1) mm. This type of waveguide has been shown to exhibit the lowest losses among hollow-metal waveguides for frequencies in the 2—3 THz range, <3 dB/m, in the study in ref (36). For the detector we use a Golay cell mounted on motorized *x*, *y*, *z* scanning stages. The Golay cell output signal is read out by a lock-in amplifier as the in-phase component of the demodulated signal after the reference phase has been adjusted to zero out the quadrature component. It is worth noting that the amplitude signal of the lock-in should not be used in any analysis requiring integration of the total power, since the measurement noise would result in a nonzero, positive contribution and hence overestimation of the 2D integral.

The Golay cell has a large input aperture. To resolve the mode profiles, we mount a 1 mm aperture in front of the Golay cell. We choose this diameter as a compromise between using a small enough sampling aperture to resolve the mode profiles, while at the same time choosing a classically large diameter that is significantly larger than the wavelength, to minimize the effects of wave-optical phenomena such as potential interference from reflections from the aperture and scattering from the aperture edges.

As a first step, the QCL emission pattern is characterized without a waveguide. This analysis is described in Appendix B, and shows that the QCL has a half angle divergence of 9.5° and emits slightly off-axis by an angle of 2.18°. This small off-axis angle shows that QCL emission direction is very well aligned with the *z*-axis.

**Figure 7:**
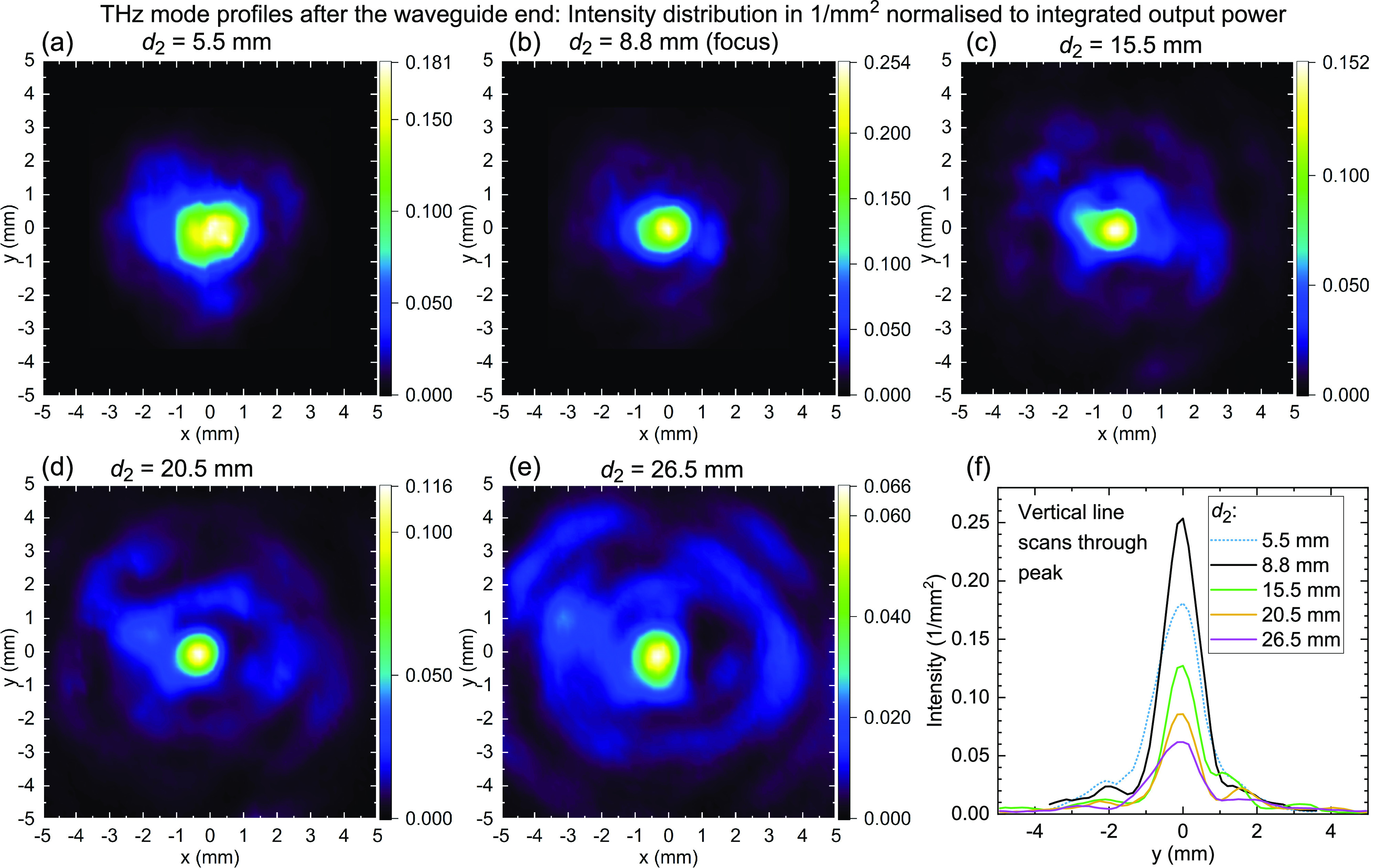
Measurements of the mode profiles at the waveguide output at different distances *d*_2_ from the waveguide end. (a–e) Intensity distributions acquired by scanning the Golay cell with a 1.0 mm iris in front of it in *x*- and *y*-directions using a 0.15 mm step size, with *L* = 238 mm and *d*_1_ = 8.9 mm. *d*_2_ = 8.8 mm corresponds to the maximum achievable central intensity, i.e., the “focal spot” of the waveguide. Closer to the waveguide end, the intensity is smaller since the power is less focused, and further away from this position, the power is redistributed into cones moving away from the waveguide axis, while the central spot becomes sharper but dimmer. In the bottom-right plot (f), vertical line scans through the peaks are shown to illustrate the peak amplitudes.

Following that, the 238 mm-long waveguide is mounted between the Golay cell detector and the QCL source, and the system is aligned. Then we measure the intensity distributions after the end of the waveguide at different z-positions, with a distance of *d*_1_ = 8.9 mm between QCL facet and waveguide, see Figure 7. Note the different color bar scales in Figure 7a–e. A quantitative comparison of the peak intensities is shown in Figure 7f depicting one-dimensional slices through the maxima in the subplots (a)–(e) along the *y*-axis.

The maximum intensity is achieved at the center, at a distance of *d*_2_ = 8.8 mm from the waveguide end. This will be called the focus or the focal spot of the waveguide. Closer to the waveguide, i.e., at lower *d*_2_, the power is less focused, i.e., the central spot is broader. This results in a lower peak intensity. For *d*_2_ > 8.8 mm, it can be seen that cones diverge away from the waveguide axis, while the peak remains at the center: The power is redistributed into the side cones moving away from the axis.

The focusing is less optimal than predicted in Figure 4f: the maximum theoretically predicted intensity is larger, and in the experiment some side rays (cones diverging from the waveguide axis) can be seen, that are absent in the theoretical prediction, Figure 6a. However, such rays are predicted theoretically, if the waveguide length is not optimal for focusing. They are clearly seen, e.g., in Figure 4d, for a slightly shorter waveguide length of 217 mm, and in Figure 6b, where the expected output profile is plotted in a cross-section for a 50 mm waveguide length. Given the strong dependence of the focal position on the waveguide radius, any deviations of the waveguide geometry from an ideal cylindrical tube will disturb the required phase coherence and reduce the quality of the focus. This may be due to variations of the radius along the length of the metallic tube, or of the geometry (e.g., ellipsoidal rather than circular cross-section). Indeed, such imperfections can be seen in Figure 3 and result in a reduced focusing strength of the real waveguide.

**Figure 8:**
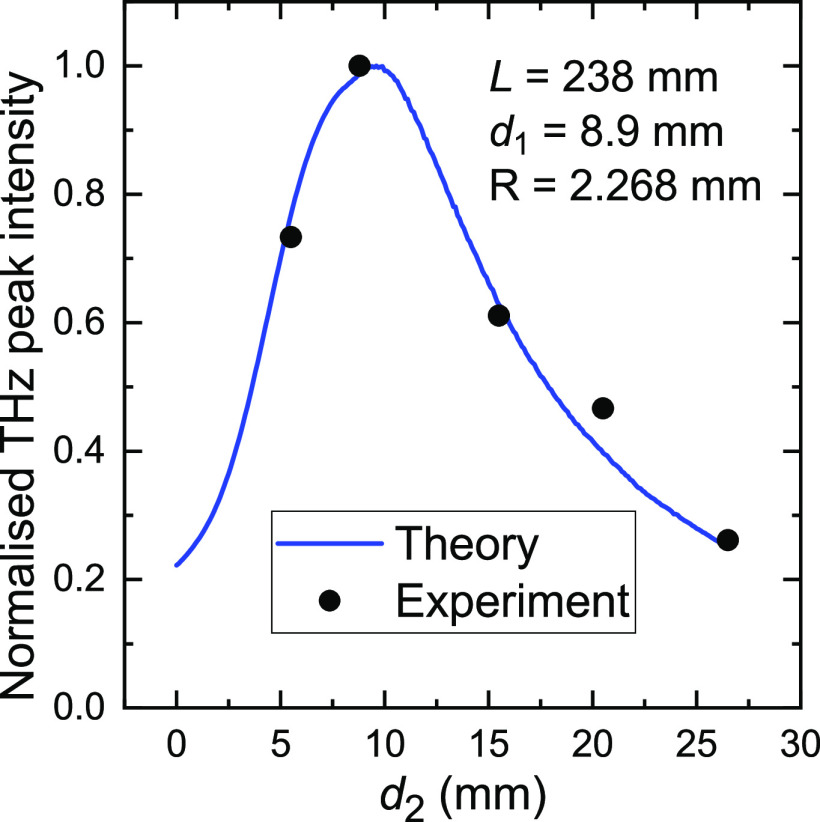
Focusing in the *z*-direction. The experimentally measured maximum intensities from Figure 7 are shown as black circles. They are compared with the theoretically predicted maximum peak intensity in dependence on *d*_2_. Both data plots are normalized to a peak intensity of 1. A good agreement of the theoretical and experimental focusing data is observed.

In Figure 8, we show the focusing effect as a function of the coordinate along the waveguide axis. The blue curve shows the theoretically predicted peak intensity as a function *d*_2_ from the ray-optical model. For comparison, the experimental maximum intensities observed in Figure 7 are shown as black circles. Both data plots in dependence on *d*_2_ are normalized to a maximum of 1. The data clearly shows that the maximum intensity is achieved at a distance *d*_2_ ≈ 8.8 mm from the waveguide end, significantly larger than the waveguide radius. The reduction in intensity before and after the “focal spot” is in good agreement with the theoretical prediction.

**Figure 9:**
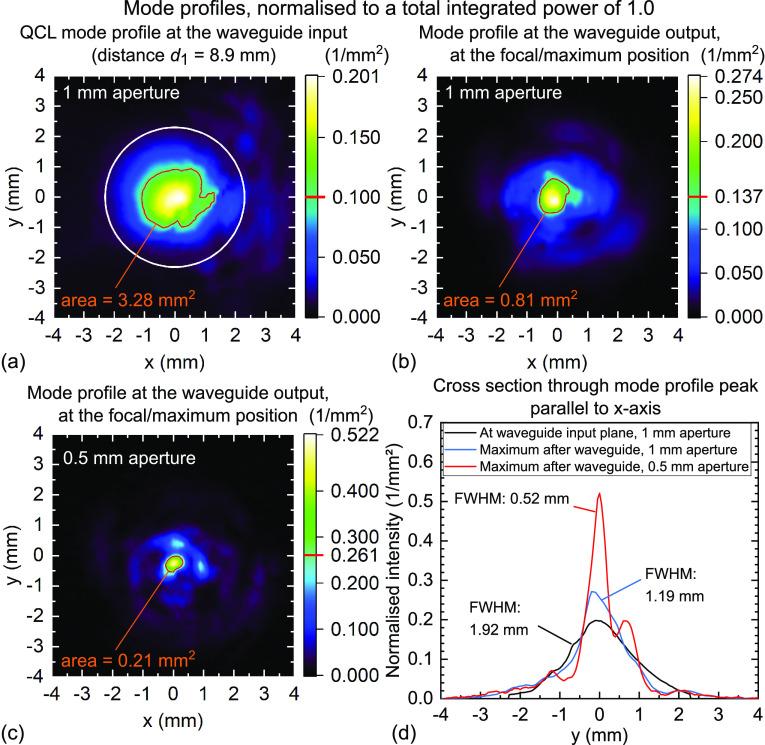
Experimental proof of the focusing ability of cylindrical multimode waveguides. Mode profiles at the waveguide input plane (a) and at the optimal focus position after the end of the waveguide (b), (c). The intensities are normalized to a value of 1.0 of the total integrated power, note the different scaling of the color bars. The red contour line is plotted at half the maximum value, and the area contained within the contour line is indicated. (a) Mode profile of the QCL before the waveguide, measured in the plane of the waveguide input at *d*_1_ = 8.9 mm with a 1 mm aperture. The white circle shows the waveguide input aperture. (b) Mode profile after the waveguide end scanned in *x*-*y*-directions at the *z*-position where the maximum intensity is observed with a 1 mm aperture, at *d*_2_ = 8.8 mm. (c) Mode profile after the waveguide end scanned in *x*-*y*-directions at the *z*-position where the maximum intensity is observed with a 0.5 mm aperture, at *d*_2_ = 5.6 mm. The Golay cell scanning step size is 0.15 mm in (a)–(c), except for the *y*-direction in plot (c), where the *y*-step size is 0.05 mm. (d) A one-dimensional plot of the vertical slices through the peaks in (a)–(c) parallel to the *y*-axis, showing the comparison of the full widths at half-maximum extracted from a Gaussian fit to the central peaks.

To conclusively prove that a multimode cylindrical waveguide has a focusing effect, the mode profile at the output of the waveguide has to be compared with that at the input of the waveguide. This is illustrated in Figure 9.

In Figure 9a, the QCL beam profile is shown in the plane of the waveguide input, at *d*_1_ = 8.9 mm. The white circle indicates the diameter of the waveguide, i.e., its input aperture. The power of the QCL contained within this circle will enter and propagate through the waveguide. In Figure 9b, the mode profile after the waveguide from Figure 7 is shown at the *z*-position where the central peak reaches maximum intensity. Both data sets were measured with a 1 mm aperture in front of the Golay cell and were normalized to a total integrated power of 1.0.

It is evident from the 2D plot that after traveling through the waveguide, the THz beam spot becomes much tighter. The mode profiles in Figure 9 have a red contour line drawn at half of the maximum observed intensity, and the area contained within the contour line is indicated. From the ratio of the areas we see that the output spot is compressed by a factor of 4. In Figure 9d, a one-dimensional cross-section along the *y*-axis through the peak is shown. A fit with a Gaussian function shows that the FWHM is reduced by a factor of more than 1.6, from 1.92 to 1.19 mm. The peak intensity has increased from 0.20/mm^2^ to 0.274/mm^2^.

The FWHM of 1.19 mm in Figure 9d is marginally larger than the 1 mm diameter of the aperture in front of the Golay cell used to sample the mode profile. This suggests that the main contribution to the peak width arises from the point spread function of the Golay cell aperture. To check this, we now put a 0.5 mm aperture in front of the Golay cell, and scan again the mode profiles. We find that with this aperture we measure the maximum peak intensity when scanning the Golay cell at *d*_2_ = 5.6 mm after the end of the waveguide. This is purely an effect of the imperfections of the waveguide: due to the deviations of the waveguide from an ideal cylindrical geometry, not all rays cross in the same theoretically expected focal spot, resulting in aberrations similar to astigmatism in an optical system. This can lead to the maximum intensity being experimentally measured at a slightly different z-position, depending on the aperture used; the beam pattern itself stays the same. The intensity distribution at this point is shown in Figure 9c. The central peak is much sharper and has an even higher normalized intensity of 0.522/mm^2^. This intensity is >8.4× higher than the expected mean intensity averaged over the waveguide cross-sectional area π*R*^2^, if an equal intensity across the cross-section is assumed. A fit to the main peak in the cross-sectional plot in Figure 9d shows that the FWHM is now reduced even further, to 0.52 mm. A comparison of the area within the half-maximum contour lines shows that the output spot is compressed by a factor >15 compared to the broad input spot. If the area within the contour line is compared to the waveguide cross-sectional area, we find that an intensity of half or more of the peak intensity is present in only 1.3% of the waveguide cross-sectional area, even at a distance of 5.6 mm after the waveguide. The actual focusing effect could be even stronger than illustrated in Figure 9.

The data underlying Figure 9a,b also allows the calculation of the waveguide power loss, which is described in more detail in the first section of the Supporting Information. The total input power entering the waveguide is quantified by integrating the unnormalized intensity within the white circle, representing the inner diameter of the waveguide, over *x* and *y*. The output power is measured by integrating the full mode profile over *x* and *y*, over the entire ±5 mm measured area, as shown in Figures S1 and S2. This allows determination of the power loss that the THz radiation encounters while traveling through the 238 mm long waveguide. From the ratio of the 2D integrals, the transmission of the THz waveguide can be estimated as 81.3%. This ray-optical approach does not consider potential reflections/scattering at the waveguide edges due to mode overlap integrals, but is justified due to the multimode nature of the large-diameter waveguide used. In the second section of the Supporting Information, we also show how the 0.5 mm aperture measurement would look like if it were captured by a 1 mm aperture, by convolving the 0.5 mm aperture measurement with an appropriate Gaussian. The difference of thus obtained intensity distribution to the experimental 1 mm aperture measurement is very small, which reinforces the validity of our approach of using a 1 mm aperture in the measurements and a Gaussian averaging in the analytical analysis, see Figure S3.

Even considering the power loss along the waveguide, the output intensity is substantially larger than the input intensity, by a factor of 1.1 in the 1 mm aperture measurement and 2.1 in the 0.5 mm aperture measurement. These results prove that the waveguide acts indeed as a focusing element, redirecting most of the input power toward its axis.

## Discussion

In the ray-optical model, Figure 5, we can see that the wavefield is approximately reproduced after a large waveguide length ≈516 mm. In the case of a symmetrical axial excitation as considered here, distinct maxima occur along the waveguide axis at halves and quarters of the full period, at a distance that we will label *T*.

In this case, the waveguide length, *L* = 238 mm, was chosen such that the focal spot is at the second maximum along the waveguide axis, as shown in Figure 6. Experimentally, the distance *T* should correspond to the total distance between the QCL source and the “focal point” after the waveguide, i.e.
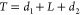
12and in our experiment, *d*_1_ + *L* + *d*_2_ = 255.7 mm. Taking the values *d*_1_ and *L* as given, the “focal length” *d*_2_ after the waveguide, where a maximum is expected, can be calculated given the results of the ray-optical model as
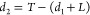
13

Let us now compare the period predicted from our ray-optical model with the results of a wave-optical calculation of the Talbot effect in cylindrical waveguides. In ref (34), the Talbot distance, i.e., the waveguide length, after which an electric field profile within an (infinite) cylindrical waveguide is approximately reproduced, has been calculated theoretically to be 16*R*^2^/λ. For our values, this distance corresponds to ≈516.4 mm. It can be clearly seen that the period observed from results of the ray-optical model employed in this work, indicated by the dashed white lines in Figure 5, is in excellent agreement with the Talbot distance derived from a wave-optical approach, which reinforces the validity of our theory. Our model enables additionally the calculation of a more general case of a cylindrical waveguide of finite length *L* with finite distances *d*_1_ and *d*_2_ of source and detector to the respective waveguide ends, which is often relevant in experiments. Thanks to the ray-optical approach we are able to calculate not only the radiation propagation within the waveguide and its period, but also the mode profiles after the end of the cylindrical multimode waveguide.

For a symmetrical excitation, the maxima along the waveguide axis occur at integer multiples of a smaller distance, which allows us to define *T* as
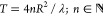
14

For the odd *n*-numbers, the achievable intensity is smaller, hence, our experiment was conducted for the second maximum (*n* = 2) with *T* = 8*R*^2^/λ.

Theoretically, for *R* = 2.268 mm and a wavelength of 159.38 μm, *T* = 258.2 mm. With *d*_1_ = 8.9 mm and *L* = 238 mm, this gives *d*_2_ = 11.3 mm. The deviation of *T* to the experimentally measured distance of *d*_1_ + *L* + *d*_2_ = 255.7 mm is very small.

It should be noted that *d*_2_ is very sensitive to small changes in wavelength λ and, particularly, of the waveguide radius *R*, that *T* has a quadratic dependence on. Experimentally, the inner waveguide diameter is known to be (4.6 ± 0.1) mm. For example, for a radius of 2.3 mm, *d*_2_ = 8*R*^2^/λ – *d*_1_ – *L* = 18.6 mm. This means, a change in radius of 32 μm, about a fifth of the wavelength, is amplified by (18.6 – 11.3 mm)/32 μm ≈ 228, and increases the focal length *d*_2_ after the waveguide by nearly two-thirds. The sensitivity to *R* comes from the fact that *d*_2_ is measured from the end of the waveguide, after a long length *L*, and therefore, the experimental determination of *d*_2_ is a differential measurement according to eq 13. In our case, we know that the waveguide radius is (2.3 ± 0.05) mm. The value of *R* = 2.268 mm is within the measurement accuracy of the waveguide radius and has been found to best describe the experimentally measured focus position.

If eq 13 is resolved toward *R*, it yields
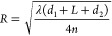
15which due to the square-root dependence of *R* on all other parameters is a well-defined problem. Such a sensitive dependence on *R* demonstrates that a measurement of the focusing position can be used as a very sensitive probe of the radius of cylindrical tubes.

The Talbot distance is scale-invariant: if *T* → *sT* and λ → *s*λ change by a scaling factor *s*, *R* → *sR* will have to change by the same scaling factor. Thus, our ray-optical model is applicable not only in the terahertz range, but for any multimode cylindrical waveguides in different regions of the electromagnetic spectrum. This way, the experimental characterization of the mode profiles after cylindrical tubes could have various applications, e.g., in the characterization of mid and far-infrared metamaterials, quality assessment of porous membranes, or inspection of micrometer-sized cylindrical tubes, needles, or capillaries with visible or UV light.

As mentioned in the introduction, in the THz range, multimode cylindrical waveguides are needed in experimental setups as a terahertz delivery system to cryogenic environments.^36,37^ Here, an approach is often used where a long waveguide system is constructed from multiple individual waveguide sections with thermal separators in-between, thus breaking the continuity of the waveguide to prevent thermal short-circuiting.^37,41^ Our ray-optical approach could be used to optimize such systems for maximum power transmission and analyze the performance of the system across the desired frequency range. The break between two waveguides should be placed at a point where most of the power is contained in modes close to the waveguide axis, to minimize losses of modes close to the edge of the waveguide. Another application lies in the design of systems where the waveguide diameter needs to be changed abruptly from one size to another. Here, the ray-optical model can be used to calculate the optimal waveguide lengths needed for minimal losses and reflections for a waveguide cross-sectional adapter.

By making use of the focusing effect of the cylindrical waveguide, we are able to combine the low losses of the multimode waveguide thanks to its large diameter (4.6 mm ≫ λ) with the ability to achieve a tightly focused spot at its output with a low divergence. The waveguide confines the light to the desired direction of propagation, and limits the amount of off-axis deviation caused by misalignment. This combination of features is more difficult to realize in a free-space setup encompassing parabolic mirrors or lenses: a low divergence requires a low numerical aperture and thus long focal length. This makes focusing to a tight spot difficult, and errors in alignment will accumulate over the length of the optical path. In addition, a waveguide solution will be likely cheaper, as one waveguide and its holder can replace two parabolic mirrors, their optical posts, holders, and tip-tilt stages. Finally, a waveguide delivery system can be very efficiently purged with nitrogen, if atmospheric absorption lines are an issue, since the only volume to be purged is the inner volume of the waveguide, in contrast to much larger volumes of free-space setups containing parabolic mirrors and lenses.

In conclusion, we predicted theoretically and proved experimentally that multimode cylindrical waveguides can be used to focus radiation without any additional optical elements such as lenses or parabolic mirrors. We described the propagation of waves within and after a multimode waveguide using a fully analytical, ray-optical model. A multimode waveguide represents an interferometric device, and the output mode profile can be understood as an interferogram of the input beam in two dimensions. The physical origin of the effect lies in self-imaging in waveguides, sometimes also referred to as the spatial Talbot effect, which we have realized for the first time for spatial focusing at terahertz frequencies after the end of a cylindrical waveguide. The presented theoretical model will allow deterministic design of waveguided systems and analysis of the output modes of cylindrical tubes. It can be readily used for the design of focusing elements in terahertz technology and radiation delivery systems, including to environments which have previously been inaccessible with free-space optics, and has direct relevance for the characterization and quality assessment of porous membranes, metasurfaces, capillaries, tubes, and photonic crystals.

## Figures and Tables

**Figure 10:**
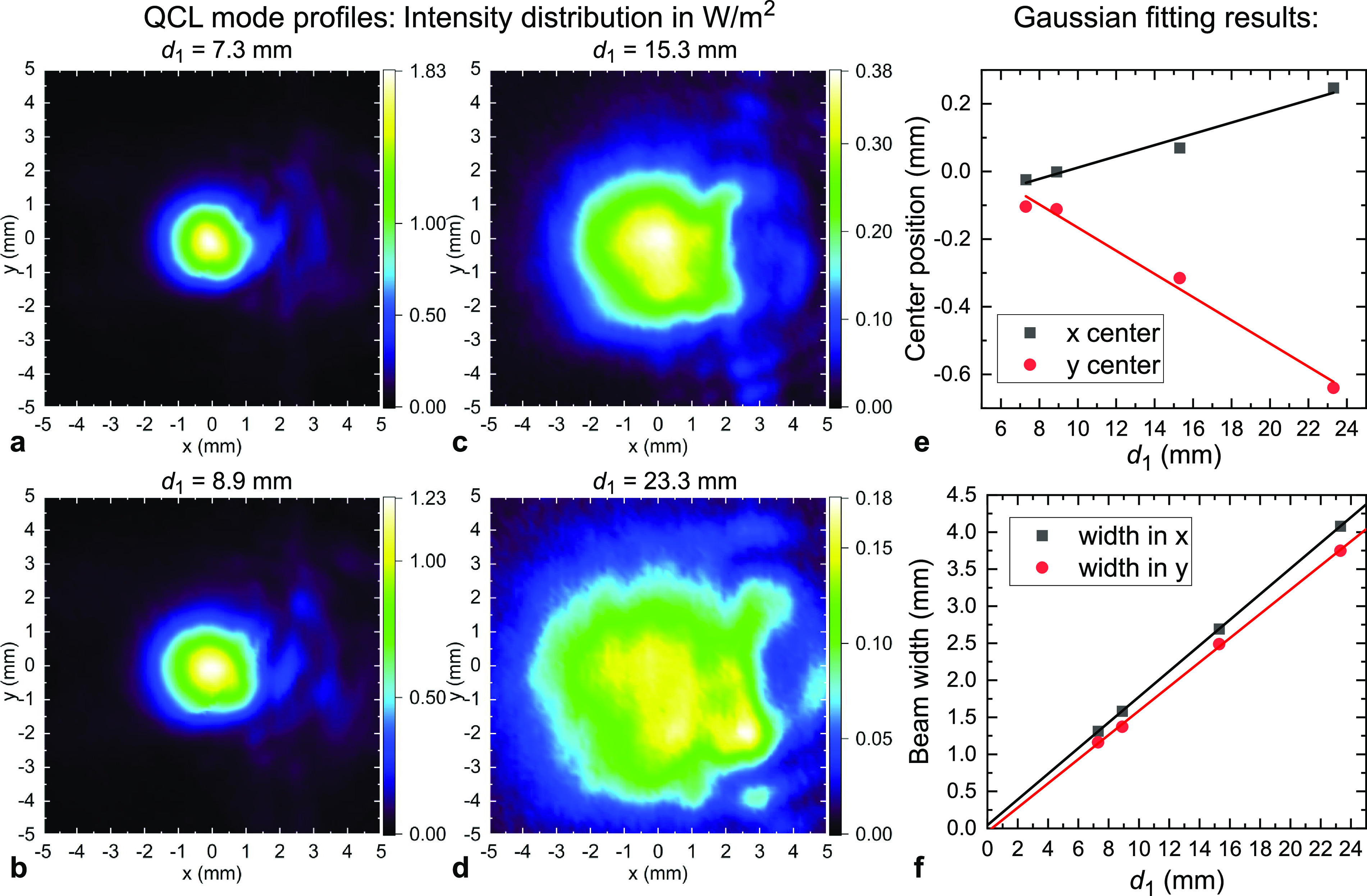
Far field characterization of the QCL emission. (a–d) Intensity distributions in W/m^2^ at different distances *d*_1_ between 1 mm iris at the Golay cell and QCL facet, measured with 0.15 mm Golay cell step size in the *x*- and *y*-directions. An almost Gaussian beam spot at small distances from the QCL widens up and loses intensity as *d*_1_ increases. (e, f) Analysis of two-dimensional Gaussian fits to the mode profiles in (a)–(d). The center position (e) and the width of the spot (f) along the *x*- and *y*-directions are shown as a function of the distance *d*_1_ between the QCL and the Golay cell aperture.

## Appendix A

### One-Dimensional Averaging

In the case of cylindrical symmetry of the mode profile, *P*(*x*, *y*) = *P*(*r*), the 2D-convolution in eqs 10 and 11 can be transformed into a 1D-convolution along the radial coordinate. Using

16

eq 11 becomes

17

By substituting ϕ′ – ϕ = χ, using the 2π-periodicity in χ and symmetry around χ = 0, it becomes clear that the integral over χ is the modified Bessel function of the first kind, *I*_0_:

18

Equation 18 allows calculation of the convolved mode profile  in the case of cylindrical symmetry.

To plot the mode evolution in a cross-section parallel to the *z*-axis, eq 18 is used to average the calculated data of *I*(*x*, *y*) along the *x*- or *y*-axes. In the case of a cylindrically symmetric mode profile, the averaging is exact and power-conserving. Here the mode profile has some weak angular dependence on the angle ϕ, hence it is used as an approximation to visualise the mode evolution in the *x*–*z*- and *y*–*z*-planes and establish periodicities, without claim of absolute numerical accuracy. However, in all the plots as a function of x and y, perpendicular to the waveguide axis, the exact 2D-convolution in eqs 10–11 is used.

## Appendix B

### Characterisation of the QCL Source

The analysis of the mode profiles requires knowledge of the source properties, such as the divergence angle of the quantum cascade laser. To answer these questions, the far field emission pattern of the QCL is measured. This is carried out by scanning a Golay cell mounted on *x*, *y*, and *z* motorized scanning stages with an iris of 1 mm diameter in front of it. Multiple *x*–*y* scans are carried out at different distances from the QCL in the *z*-direction, as shown in Figure 10a–d. To quantify the QCL emission pattern, the mode profiles are fitted with a two-dimensional Gaussian function:

19

This allows extraction of the center positions *x*_c_, *y*_c_ and beam widths *w*_*x*_, *w*_*y*_ along the *x*- and *y*-directions, which are shown in Figure 10e,f. By fitting values for *w*_*x*_ and *w*_*y*_ as a function of *z*, we find that the slopes of the linear fits to the width data are 0.1731 in the *x*-direction and 0.1634 in the *y*-direction. By setting the slope equal to tan α, we deternine the divergence half-angles of the QCL beam as α_*x*_ = 9.8° and α_*y*_ = 9.3°. We use an average value of α = 9.5° in the theoretical calculations.

The center positions of the beam spots indicate how much the propagating beam moves away from the *z*-axis and can be used to determine the misalignment angles. The slope of the linear fits to the center positions are 0.0171 in the *x*-direction and −0.0351 in the *y*-direction. This defines the misalignment of θ_0_ = 2.18° with respect to the *z*-axis, at a polar angle of ϕ_0_ = −64° (measured with respect to the positive *x*-axis).

## Supporting Information Available

Waveguide power loss methodology; Comparison of 0.5 mm and 1.0 mm aperture; Figures S1–S3 (PDF)
