# Supplementary material for: Cylindrical Multimode Waveguides as Focusing Interferometric Systems
Source: ACS Photonics. 2023 May 17;10(6):1756–68. doi: 10.1021/acsphotonics.2c02030 (PMC10288537; doi:10.1021/acsphotonics.2c02030)
Supplement: Supplementary file 1 — ph2c02030_si_001.pdf [file ph2c02030_si_001.pdf]

Supporting information to: Cylindrical multimode waveguides as focusing interferometric systems

Wladislaw Michailow,* Nikita W. Almond, Harvey E. Beere, and David A. Ritchie

Cavendish Laboratory, University of Cambridge, JJ Thomson Avenue, CB3 0HE Cambridge, UK

E-mail: wm297@cam.ac.uk

6 pages with 3 figures, Figs. S1–S3.

1 Waveguide power loss methodology

Here we explain how we estimated the transmission of the waveguide, stated as 81.3% in the main text.

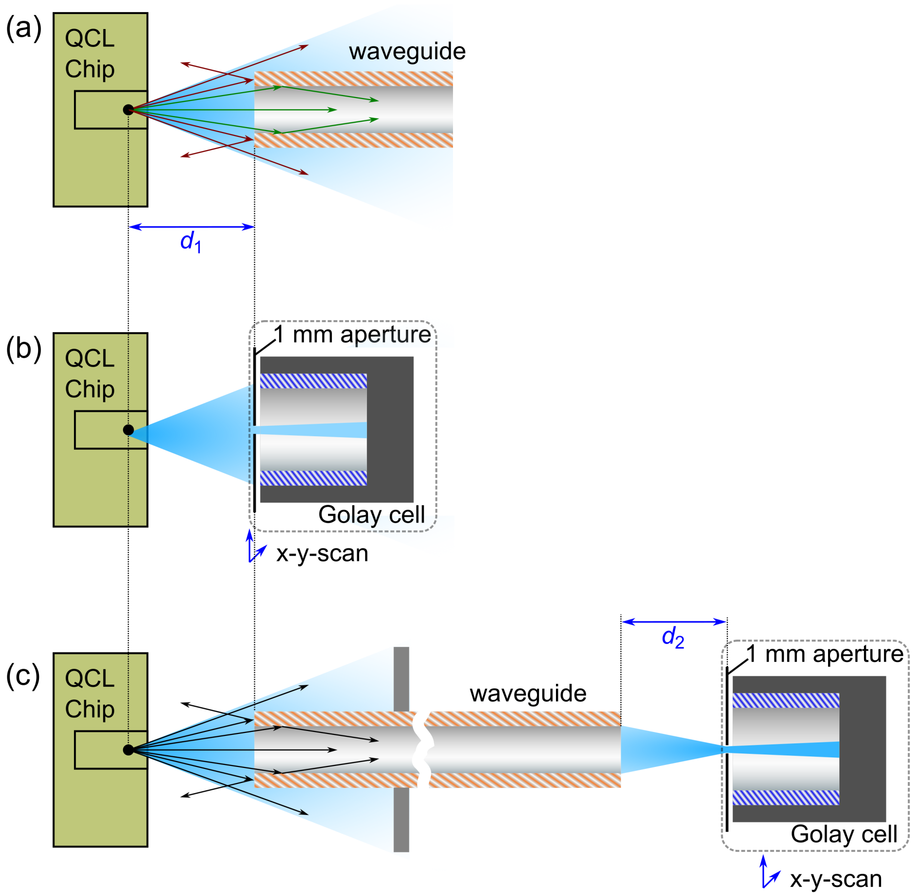

Figure S1: **Schematic diagrams for the measurement of the waveguide transmission loss.** (a) Rays that enter the waveguide (dark green) and that miss the waveguide entrance (dark red). (b) Measurement of the QCL beam pattern at the entrance of the waveguide. (c) Measurement of the mode profile at d_2 after the waveguide.

As shown in Fig. S1 (a), rays entering the waveguide from the QCL have three possible paths: they enter the waveguide (shown as dark green arrows), or they are reflected back at the front face of the waveguide, or they miss the waveguide and follow a straight path into free space. The latter two beam paths are illustrated by dark red arrows.

The 4.6 mm inner diameter waveguide used in the experiment has an outer diameter of approximately 6.3 mm. For our power loss analysis, we aim to determine by how much the

power of the radiation that has entered the waveguide is attenuated by the time it reaches the other end. This means that only the power contained in the rays indicated by the dark green color in Fig. S1 (a) should be considered as the power that entered the waveguide, used as a reference for the transmission calculation. This allows us to exclude sample- and setup-specific parameters, such as the width of the waveguide walls or the angle of divergence of the QCL emission. We assume that all dark green rays that travel toward the hollow cylindrical inner part of the waveguide enter the waveguide, and neglect wave-optical scattering on the circular edge of the waveguide. Because the diameter of the waveguide, 4.6 mm, is much larger than the radiation wavelength of 159.38 µm, this ray-optical approach is justified.

For this characterisation, we measure the full mode profile at the "focus" position $d_2 = 8.8\,\mathrm{mm}$, as illustrated in Fig. S1 (c), by scanning the Golay cell with a 1 mm aperture in the x and y lateral directions. Then we remove the waveguide and repeat the scanning by placing the Golay cell directly in front of the QCL. The distance between 1 mm aperture and QCL is set to the same distance as d_1 used previously for the measurements with the waveguide. The 1 mm aperture used for this characterisation is a flat, fixed-diameter aperture fabricated from a copper sheet, that was not changed or adjusted in any way between the measurements shown in Fig. S2 (a) and (b) to ensure comparability.

The result of the two measurements is shown in Fig. S2. The data in this figure is from the same measurements as presented in Fig. 9 (a), (b) in the main text, except that here the data is not normalised to the integrated power. Instead the original signal is shown in units of millivolts, measured as the output voltage of the Golay cell, captured by the sign-sensitive in-phase component at the output of a lock-in amplifier. This allows quantitative comparison of both measurements.

The obtain the full power transmitted through the waveguide, we take a 2D integral of the data shown in Fig. S2 (b), which yields $17.8\,\mathrm{mV{\cdot}mm^2}$. To obtain the full power that entered the waveguide, we take a 2D integral of the intensity shown in Fig. S2 that is contained within a circle of 4.6 mm diameter around the center, shown by a dotted white

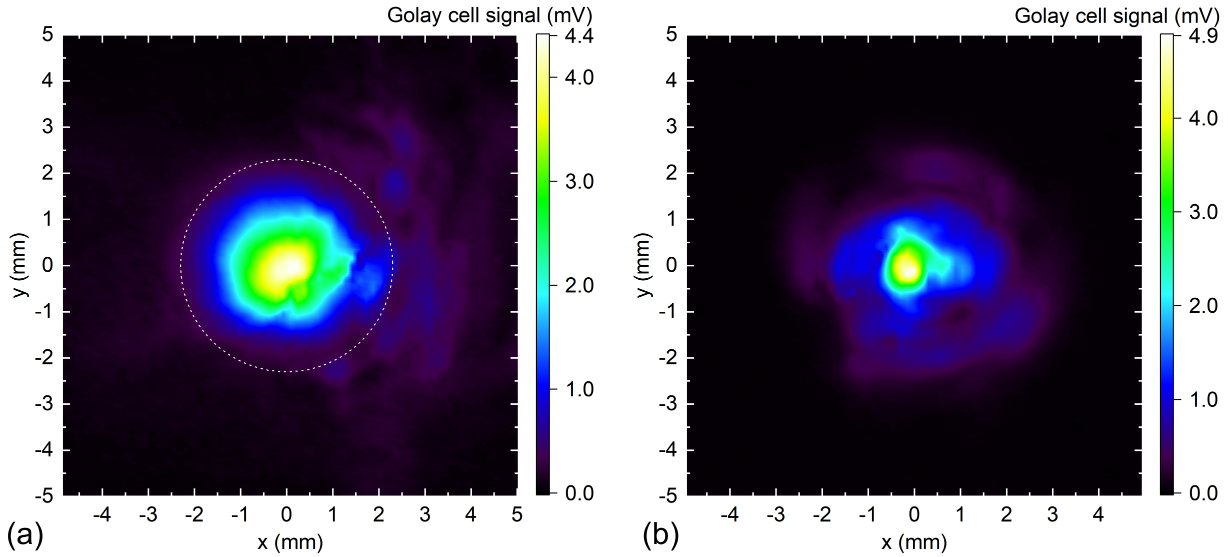

Figure S2: **Data underlying the waveguide loss characterisation.** Mode profiles at the waveguide input plane (a) and at the optimal focus position after the end of the waveguide (b), measured as Golay cell output voltage in millivolts. The Golay cell scanning step size is 0.15 mm. (a) Mode profile of the QCL before the waveguide, measured in the plane of the waveguide input at $d_1 = 8.9$ mm with a 1 mm aperture. The white circle shows the waveguide input aperture. (b) Mode profile after the waveguide end scanned in x-y-directions at the z-position where the maximum intensity is observed with a 1 mm aperture, at $d_2 = 8.8$ mm.

line that indicates the radial cut-off for the integration. This gives a value of 21.9 mV·mm^2. The obtained power ratio is 81.3 %, which is the estimated transmittance of the radiation power as it travels through the 238 mm-long waveguide.

2 Comparison of 0.5mm and 1.0mm aperture

In Fig. 9 of the main text we have demonstrated the focusing ability of a multimode cylindrical waveguide. We showed a measurement with a 1.0 mm aperture and with a 0.5 mm aperture. These measurements are also shown here in Fig. S3 (a), (b). Here we answer the question, how would the mode profile captured with a 0.5 mm aperture be transformed if it were captured with a 1.0 mm aperture?

To answer this question, we convolve the 0.5 mm aperture measurement, Fig. S3 (b), with a Gaussian function, as described in Eqs. (10)–(11) in the main text. We choose the averaging constant a in the Gaussian function $e^{-(x^2+y^2)/a^2}/(\pi a^2)$ such that the peak intensity of the convolved mode profile equals the experimentally measured peak intensity for the 1.0 mm aperture measurement in Fig. S3 (a) of $0.274/\text{mm}^2$. This is the case for an averaging constant of $a = 0.302\,\text{mm}$, corresponding to an FWHM of 0.503 mm. The result of the convolution is shown in Fig. S3 (c). It shows the convolution of the 0.5 mm aperture measurement in Fig. S3 (b) with a Gaussian with averaging constant $a = 0.302\,\text{mm}$, and can be understood as the theoretically expected mode profile that would be observed with a 1.0 mm aperture, based on measurement data of a mode profile with 0.5 mm aperture.

As can be seen, the resulting mode profile is very similar to Fig. S3 (a), the experimental measurement with a 1.0 mm aperture. Even the quantitative area enclosed by a contour line at half the peak value is the same, within 1% accuracy. The observed deviations are very small, and can be observed mainly for two reasons: firstly, the two measurements were carried out at slightly different distances $d_2 = 8.8\,\text{mm}$ in Fig. S3 (a) vs. $d_2 = 5.6\,\text{mm}$ in Fig. S3 (b), resulting in a small change of the overall mode profile; and secondly, the Gaussian function is used as an approximation of the true point spread function of the aperture. Overall the agreement between Fig. S3 (a) and (c) is very good, which reinforces the validity of our approach of using a 1 mm aperture in the measurements and a Gaussian averaging in the analytical analysis.

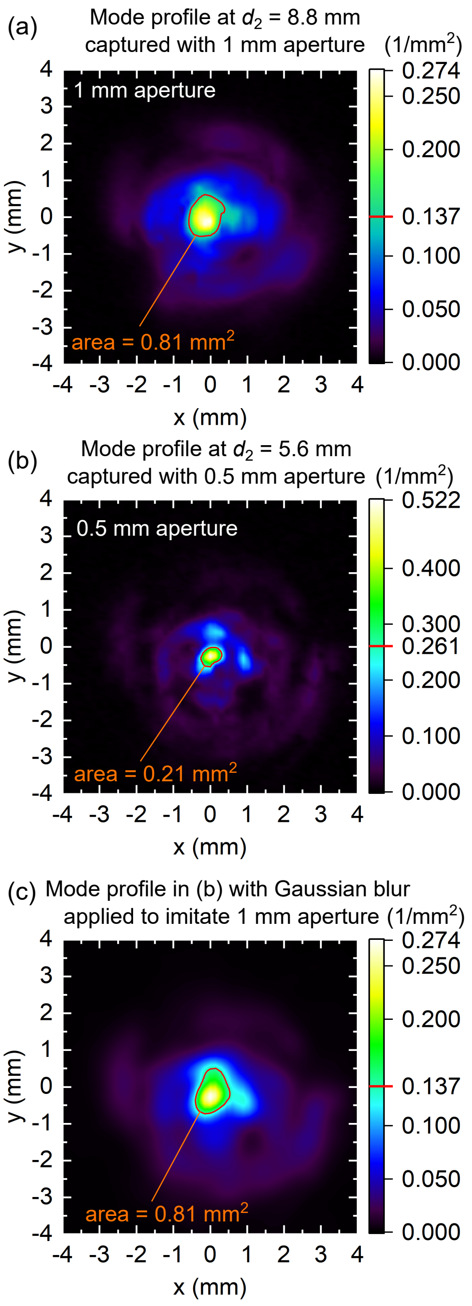

Figure S3: **Influence of aperture size on measurement.** (a), (b): Copy of Fig. 9 (b), (c) in the main text; mode profiles at the waveguide output, scanned in x-y-directions at the z-position where the maximum intensity is observed. (a) Measurement with a 1 mm aperture, at $d_2 = 8.8$ mm. (b) Measurement with a 0.5 mm aperture, at $d_2 = 5.6$ mm. (c) Convolution of the measurement in (b) with a Gaussian with averaging constant $a = 0.302$ mm.
